# Dietary Behaviour and Sociocultural Determinants of Dietary Diversity among Rural Women of Reproductive Age: A Case of Amhara Region, Ethiopia

**DOI:** 10.3390/nu15153369

**Published:** 2023-07-28

**Authors:** Simegn Kassa Alamirew, Stefanie Lemke, Barbara Stadlmayr, Bernhard Freyer

**Affiliations:** 1 Institute of Development Research (IDR), University of Natural Resources and Life Sciences, Vienna, 1190 Vienna, Austria; stefanie.lemke@boku.ac.at (S.L.); barbara.stadlmayr@boku.ac.at (B.S.); 2 Division of Organic Farming, University of Natural Resources and Life Sciences, Vienna, 1190 Vienna, Austria; bernhard.freyer@boku.ac.at

**Keywords:** dietary behaviour, dietary diversity, women of reproductive age, sociodemographic and sociocultural determinants, Ethiopia

## Abstract

Women of reproductive age have specific nutritional requirements due to pregnancy and lactation. Little is known about the sociocultural determinants of dietary diversity among women of reproductive age. This study assesses trends of dietary behaviour and associated determinants of dietary diversity of women of reproductive age. A community-based cross-sectional study was conducted in the Amhara region of Ethiopia in 2019. Using multistage systematic random sampling, the dietary diversity of *n* = 421 women of reproductive age was assessed by a qualitative 24 h dietary recall. Descriptive analysis revealed characteristics of dietary behaviour and a chi-square test enabled the identification of associated determinants of women’s dietary diversity. Only about a quarter (26.8%) of the women consumed five or more food groups per day and met the minimum dietary diversity score (MDD-W). Drawing on the socioecological framework, at an intrapersonal/individual level, women’s education, age, perception of nutritious diet, and frequency of consumption of animal-sourced foods, vegetables, and fruit were significantly associated with MDD-W. At an interpersonal/household level, the husbands’ education, women’s decision-making regarding food purchase/consumption, the family’s actual eating occasion, and women’s engagement in domestic and farming tasks were significantly associated with MDD-W. At a community level, access to clean water and especially cultural beliefs were significant determinants of MDD-W. Amharic proverbs and sayings prioritise men and pose severe restrictions on women regarding food allocation. The majority (76.7%) of women of reproductive age practise frequent religious fasting, relating to the institutional/national level. This undermines efforts to support healthy dietary behaviour of women of reproductive age. Indepth studies on religious and cultural practices are needed, to assess not only their negative effects on the dietary diversity of women of reproductive age but also on women’s lives.

## 1. Introduction

Dietary behaviour is an umbrella term referring to all phenomena related to food choice, eating habits, and dietary intake [1]. Within the concept of dietary behaviour, this study investigates the characteristics of dietary diversity, eating habits, and perceptions regarding nutrition of women of reproductive age (WRA), i.e., 15–49 years, in rural areas in the Amhara region, Ethiopia.

Women of reproductive age are often nutritionally vulnerable due to the biological demands of pregnancy and lactation [2]. Maternal micronutrient deficiencies lead to widespread nutrition challenges that affect not only women but also their children [3,4]. These negative effects include the overall risk of mortality and a variety of adverse health effects, including poor cognitive development, decreased immunity, and impaired work capacity. During pregnancy, micronutrient deficiency increases the risk of low birth weight, miscarriage, fetal malformations, and child mortality [3,5,6].

Low- and middle-income countries, including in sub-Saharan Africa, are among the regions with the highest burden of micronutrient deficiencies among WRAs [7]. According to a recent review across four sub-Saharan countries, including Ethiopia, Kenya, Nigeria, and South Africa, the prevalence of the most common micronutrient deficiencies among WRAs ranged up to 51% for iron, 22% for vitamin A, 55% for iodine, 34% for zinc, and 46% for folate [8]. While micronutrient deficiencies and undernutrition persist in sub-Saharan Africa, overweight and obesity are also rapidly increasing, particularly among adolescent girls and adult women, with a prevalence of overweight ranging from 29.6% in Rwanda to 34.8% in Tanzania in 2018 [9]. Malnutrition in all its forms (i.e., undernutrition, micronutrient deficiencies, and overweight/obesity) is also a public health problem in Ethiopia [5,10]. A recent study in the Arsi zone, Oromo region, indicates that almost half (48.6%) of WRAs are affected by malnutrition [11]. Levels of undernutrition are high, with 21% of WRA being affected in Ethiopia and 24.8% of WRAs being affected in the Amhara region [12]. A recent study in the South Wollo Zone, Amhara region, found that the prevalence of anaemia is 24% among WRAs [13]. Evidence from Dire Dawa, Eastern Ethiopia, showed that being overweight affects up to 63.1% of WRAs [14].

Poor dietary intake, such as a low intake of fruit, vegetables and whole grains and a high intake of red and processed meats, are among the leading causes of health problems and mortality [4,15]. Thus, the consumption of a diversified diet and adequate micronutrient intake contributes to preventing premature adult mortality [15]. It is estimated that dietary diversity could reduce by up to 22% of all diet-related premature deaths among adults in Africa [4].

Dietary behaviour is associated with a range of individual, social, and cultural determinants [16,17,18,19]. In Ethiopia, studies that assess sociocultural practices as determinants of inadequate minimum dietary diversity of women (MDD-W) are limited. Several studies focus on individual-level and sociodemographic determinants [20,21,22,23]. Other studies report that religious fasting [24,25] and taboo foods [26,27,28,29] are determinants of inadequate dietary practice for pregnant women in Ethiopia. This shows that, apart from religion and taboo food practice, other social and cultural aspects that might influence the dietary behaviour of WRA are not assessed.

In 2016 the government of Ethiopia launched the “Seqota” Declaration, with the goal of eliminating all forms of malnutrition among children under two years of age by 2030, with programmes targeted at improving the health and nutritional status of women, children under two years of age, and adolescent girls [30]. However, this goal cannot be achieved unless sociocultural aspects, including gender inequality related to dietary behaviour, are addressed in Ethiopia.

Cultural practices are linked to the identity of societies, to a certain extent governing behaviours and actions [31,32]. As every society has its own specific food culture related to the perceptions and practices of when, what, and how to prepare and consume foods, social norms can influence the type of foods individuals in a particular community consume [33,34].

According to our knowledge, there is no study that investigated sociocultural determinants, including the use of Amharic proverbs and sayings related to food intake, to understand the dietary behaviour of WRAs in Ethiopia. The Amhara region is one of the three regions in Ethiopia with the highest number of WRAs [35]. In this region, there are still deeply rooted religious and cultural practices that could restrict women not to consume their preferred foods. The aim of this study is, therefore, to understand dietary behaviour and to identify associated sociodemographic and sociocultural determinants of dietary diversity of WRAs. The analysis is guided by drawing on the socioecological framework adapted from a recent review on dietary and physical activity behaviour of WRA in sub-Saharan Africa [18]. Our study discusses the following research questions: (i) what are the characteristics of dietary behaviour (e.g., dietary diversity practice, eating habits such as actual eating occasions within the family, cultural and religious eating habits, and perception of nutritious diet) of WRAs? (ii) Which determinants at the intrapersonal/individual level, interpersonal/household level, community level, and institutional level affect the dietary diversity of WRA?

## 2. Materials and Methods

### 2.1. Study Design and Conceptual Framework

**Figure 1:**
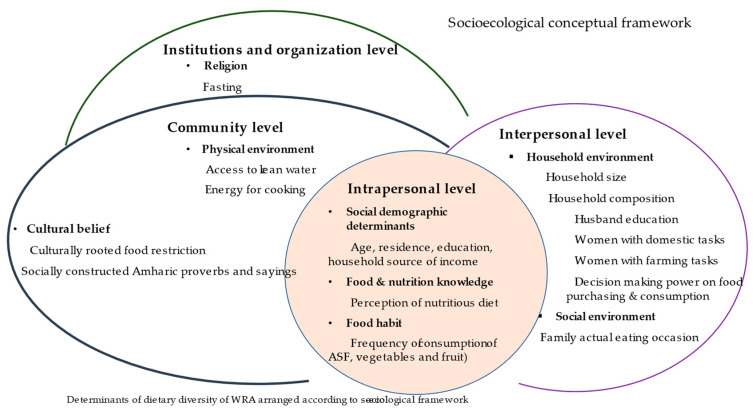
Determinants of dietary diversity of rural WRA in Amhara region Ethiopia. Socioecological framework adapted from [18].

A community-based cross-sectional study was conducted between April and August 2019 in Amhara regional state, Ethiopia. To understand the multidimensional determinants of dietary diversity of WRAs, we draw on the socioecological conceptual framework of Yiga et al. (2020), who adapted it to the African context [18]. The framework was originally developed by Bronfenbrenner in the late 1970s [36]. It enables the identification of determinants at different levels, illustrating the close interlinkage of all determinants and how they influence each other (Figure 1). For this study, we adapted the categories at each level according to our specific context, enabling us to identify and categorise determinants of dietary diversity of WRA.

The intrapersonal/individual-level determinants in our adapted framework include three categories: sociodemographic characteristics, (i.e., age, education, household source of income, and place of residence); food and nutrition knowledge, (i.e., perceptions of a nutritious diet); and food habits, (i.e., frequency of consumption of animal-source food (ASF), vegetables, and fruit).

The interpersonal/household level determinants comprise two categories: household environment and social environment. The household environment includes the household size and household composition (i.e., the husband’s education, women’s domestic and farming tasks, and women’s decision-making on food purchasing and consumption). At this level, for instance, the husband’s education may either provide social support in a household or create barriers that affect the dietary diversity of the WRA. In addition, women’s domestic and farming tasks are assessed to understand the influence of workload on the dietary diversity of WRA and women’s decision-making power in food purchasing and consumption is used to assess the influence of gender dynamics related to food consumption. The social environment includes the family’s actual eating occasion.

Community-level determinants comprise the physical environment and cultural beliefs. The physical environment includes the accessibility and availability of resources that may affect the dietary diversity of WRAs, such as access to clean water and access to energy for food preparation. Cultural beliefs result in culturally rooted food restrictions; for example, a cultural norm that expresses women should not eat certain parts of chicken and lamb meat. Further, socially constructed Amharic proverbs and sayings lead to food restrictions; for example, “prioritizing the husband in food consumption”, “women should not eat outside their house or in public places” and “women should not consume adequate amounts of food”.

Lastly, at the institutions and organisation level, we focus on religion as one dominant institution at the national level, assessing if religious status and religious food restrictions/fasting are associated with the dietary diversity of WRA.

### 2.2. Study Setting and Selection of Study Area and Sample Size

The Amhara region has an estimated total population of 21,134,988 (10,585,995 male and 10,548,993 female) according to the projection of the Central Statistical Agency (CSA) of the population in Ethiopia from 2014–2017 [37]. About 90% of the Amhara population lives in rural areas, with the majority depending on agriculture for their livelihood. The majority of the Amhara population (98%) practices the Ethiopian Orthodox Christian religion [38]. Ethiopia is administratively divided into four levels: regions, zones, woredas (districts), and kebele (wards). The Amhara region consists of 11 zones [39]. The study was conducted in the West Gojjam zone in Bahir Dar Zuria woreda (district) and Merawi (Mecha) woreda, and in the South Gondor zone in Dera woreda. West Gojjam Zone has 14 woredas with a total population of 2,542,221, of which 50.7% were female in 2017. The South Gondar zone has eleven woredas with a total population of 2,484,929, of whom 1,257,323 were men and 1,227,606 were women in 2017 [37].

**Figure 2:**
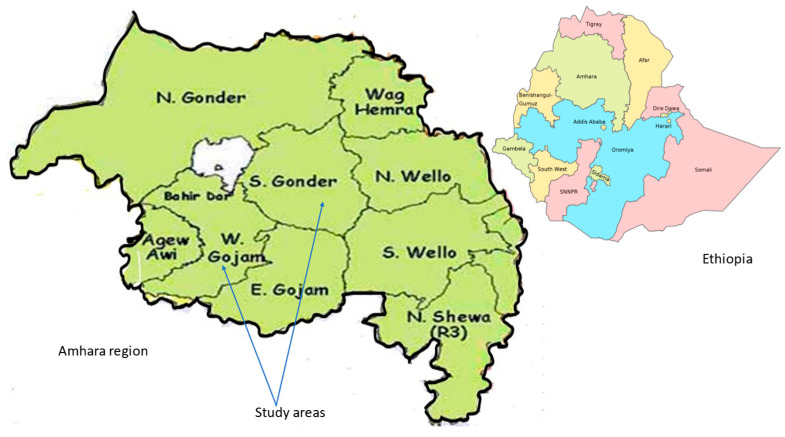
Map of Administrative zones, Amhara Region study areas. Source: https://commons.wikimedia.org/wiki/File:Ethiopia_regions_zones_administration.jpg accessed on 15 June 2023).

**Figure 3:**
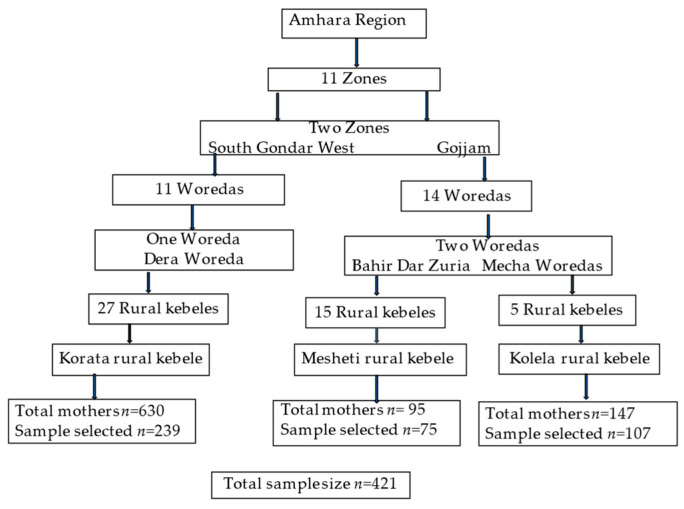
Steps for the selection of sample size. Source: the Authors.

For the selection of the study area and sample size, a multistage sampling technique was applied from the highest administrative division of the zone to the lowest administrative division of the kebele. First, all zones in the Amhara region were listed and a lottery sampling method was applied to choose two zones. At the next step, all woredas in the two selected zones were listed and a lottery sampling method was again applied to select two woredas in each zone. From each woreda, all kebeles were listed, using the lottery method, choosing one kebele in each woreda. Finally, at the kebele level, the lottery sampling method resulted in the selection of Korata rural kebele in Dera woreda, Meshenti rural kebele in Bahir dar Zuriea woreda, and Kolela rural kebele in Merawi/Mecha woreda (Figure 2 and Figure 3).

The sample size was calculated by using a single population proportion formula 95% confidence level, a 5% margin of error, and taking a 50% proportion of the reproductive age women with MDD-W. The calculated total sample size was *n* = 421 WRA across the three districts with an assumption of a 10% nonresponse rate. To reach the sample size of participating women in each kebele, a systematic random sampling method was used. Based on the recorded data that were found in each kebele health centre, the women were identified as those women who (i) had undergone a pregnancy follow-up in the health centre, (ii) did not have a history of any chronic diseases, and (iii) had children under two years of age. The total number of women who met these criteria was listed and every tenth woman was selected until the calculated total sample size was reached.

### 2.3. Data Collection Procedure

A structured questionnaire was developed in English and translated into Amharic. The questionnaire included 40 questions, seeking information on the following: sociodemographic determinants such as age, educational status, household source of income, and residence; WRA perception of a nutritious diet and malnutrition; food habits regarding the frequency of consumption of ASF, vegetables, and fruit; household environment-related factors such as household size, household composition, husband’s education, women performing domestic and farm tasks, women’s decisions on food purchasing and consumption; household social environment-related factors, namely the family’s actual eating occasion; as part of the physical environment, access to clean water, energy for cooking, and having a latrine. Sociocultural determinants were taboo food, food-related proverbs and sayings about women’s dietary diversity, and religion and religious food restriction/fasting.

Face-to-face interviews were conducted with WRAs, lasting on average 45 min. The first author (S.K.A.) was supported by six trained health extension workers who spoke Amharic and who were familiar with the specific region and context, and who were experienced in data collection. Everyone involved in data collection received a one-day training about the purpose of the study, data collection methods, and ethical issues.

Supervision of the data collection process was provided by a Master’s student at Bahir Dar University Social Anthropology Department, the principal investigator (S.K.A.), and the second scientific supervisor of this thesis who was also the principal investigator of the larger study (B.F.). Before the actual data collection started, a pilot study was conducted of 5% of the total sample size. The purpose was to assess the questionnaire for its clarity, length, and completeness. Some ambiguous sentences and terms and also questions that proved difficult to ask were rephrased. Throughout the data collection period, the collected data were checked for completeness, accuracy, and consistency.

The outcome variable “dietary diversity” was assessed using the Food and Agriculture Organisation’s (FAO) indicator of minimum dietary diversity score for women (MDD-W) [2]. The food groups are (1) grains, white roots and tubers, and plantains; (2) pulses; (3) nuts and seeds; (4) dairy; (5) beef, sheep/goat, poultry, and fish; (6) eggs; (7) dark green leafy vegetables; (8) other vitamin A-rich fruit and vegetables; (9) other vegetables; and (10) other fruit. All available foods consumed in the study area were recorded, including local dishes that were consumed for breakfast, lunch, dinner, and snacks in between, based on the above-mentioned ten food groups [4]. The women were asked to recall what they had consumed within the past 24 h. Finally, the women’s dietary diversity score (MDD-W) was calculated. Women who consumed at least five or more of the 10 food groups achieved the MDD-W and are, therefore, more likely to have adequate micronutrient intakes [2]. If women consumed fewer than five of the ten food groups, their micronutrient intake was considered inadequate.

### 2.4. Data Analysis

Data were first checked for completeness and consistency and entered into the Statistical Package for Social Science version 26 (SPSS Inc., Chicago, IL, USA). Then, the data were cleaned and explored for outliers, missing values, and any inconsistencies by visualizing, calculating frequencies, and data sorting. The visual inspection of histograms, skewness, box plots, and the homogeneity hypothesis test were used to test for the normality of the data distribution of independent variables. The symmetry McNemar chi-square test was applied to check the marginal homogeneity of variables such as the education status of women and their husbands. Corrections were made by checking the original data for further analysis. Descriptive statistics were used to calculate percentages, frequencies, and medians. Continuous variables (e.g., age, household size) are presented as the median in the text and categorical variables are presented as counts and percentages in the text and in the tables. A cross-tabulation with Pearson’s chi-square statistics was run to assess the relationship between sociodemographic and sociocultural determinants and MDD-W. *p* < 0.05 was considered to be statistically significant.

### 2.5. Ethical Considerations

Before data collection, ethical approval was obtained from the Department of Social Anthropology, Faculty of Social Sciences, Bahir Dar University. To conduct the fieldwork, an official letter in the Amharic language was sent to all health centres in the woreda/districts to obtain permission for data collection. The aim of the study was also explained to the officials of the health centres of each woreda to obtain their consent and support. All women who participated in the study were fully informed of the purpose of the study and their right to withdraw from the study at any time. Data was collected after verbal consent was obtained from study participants in their local language. The data obtained from each study participant were kept confidential.

## 3. Results

### 3.1. Sociodemographic Characteristics

A total of *n* = 421 WRA participated in this study. The median age of the respondents was 29 years, with the majority (75.3%) being below 32 years (see Table 1). The majority of WRAs were Orthodox Christian (91.4%), followed by Muslim (8.6%). Regarding education (40.6%) of the women and (31.8%) of their husbands were not able to read and write; (29.0%) of the women and (25.2%) of their husbands had attended primary education, while (9.3%) of their husbands had attended secondary school or higher education, respectively. All interviewed women stated that their husband is the head of the family and makes a higher contribution to earning household income. Household source of income refers to the income earned by the household members. More than half of WRAs (59.4%) derived a household income from agricultural production, while (36.5%) of WRAs obtained income from nonagricultural work, including minishops (i.e in their house selling small items like soap, tea, bread, oil, salt, etc.), daily off-farm labour, trade (i.e., selling goods in the nearby open-air market, i.e., agriculture products, ‘chat’, etc.) and weaving. Household size, expressed as number of children living in a household, ranged from 1 to 8 children, with a median of 3 children per household.

### 3.2. Dietary Behaviour of WRA

The dietary behaviour of WRAs in this study includes dietary diversity practice, eating habits (e.g., actual eating occasions within the family, and cultural and religious eating habits) and perceptions regarding a nutritious diet.

#### 3.2.1. Dietary Diversity Practice

**Figure 4:**
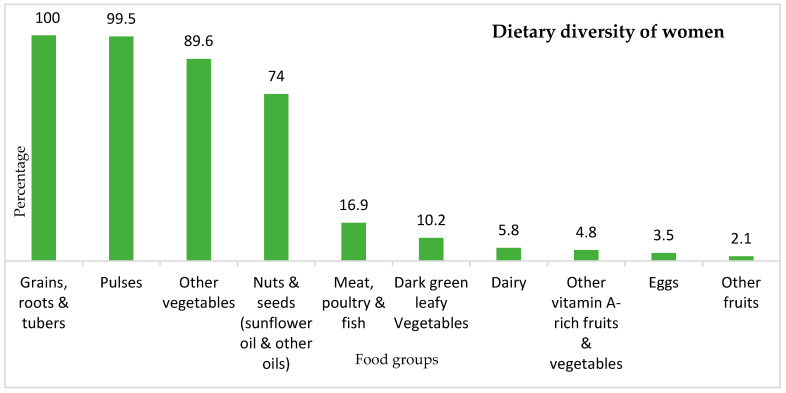
Percentage (%) of consumption of food groups by WRA (*n* = 421), Amhara region, Ethiopia 2019.

**Figure 5:**
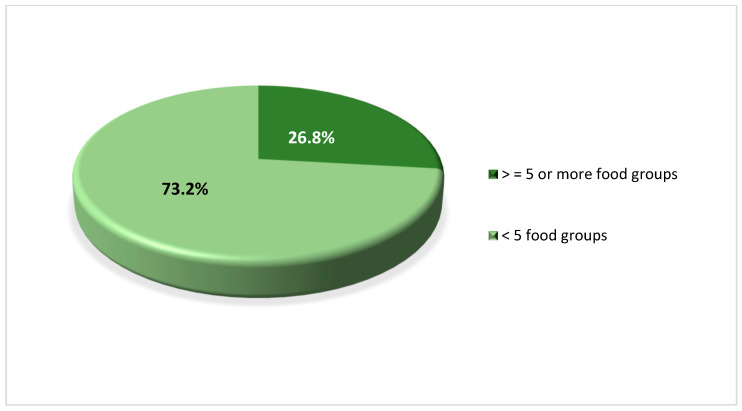
Minimum dietary diversity score of WRA in Amhara region, Ethiopia 2019 (*n* = 421). The dark green colour of the diagram indicates WRA who achieved MDD-W by consuming five food groups and above within the past 24 h while the light green colour refers to WRA who did not achieve the MDD-W as they consumed less than five food groups.

Findings on dietary diversity practice (Figure 4) indicate that WRAs predominantly consumed starchy staples, including grains (e.g., teff, an ancient grain/edible seed originating from Ethiopia which is used to make the traditional bread ‘injera’, finger millet/dagusa, maize, and wheat); roots and tubers (e.g., potato) (100%), followed by pulses (e.g., beans, peas, chickpeas, and lentils) (99.5%), and other vegetables (e.g., onions and tomatoes) (89.6%). ASF, including meat, poultry and fish (16.9%), as well as a range of other nutritious food such as dark green leafy vegetables, eggs and fruit, were least consumed. The average dietary diversity among WRAs was 4 ± 0.74 SD, ranging from two to six food groups. The majority of the women (73.2%) did not achieve the MDD-W (>5 food groups out of ten food groups) and are, therefore, less likely to have adequate micronutrient intakes. Just over a quarter (26.8%) of WRAs achieved the MDD-W and consumed five or more food groups out of the ten food groups on the previous day (Figure 5).

#### 3.2.2. Eating Habits of WRAs within the Family’s Actual Eating Occasion

More than half of WRAs (59.9%) served their families before consuming food themselves. Of these, 40.9% were uneducated (not able to read and write). More than a third of WRAs (37.8%) consumed food together with their families. Few WRAs (2.9%) consumed food before serving their families (Table 2, also for the following results presented in Section 3.2.3 and Section 3.2.4). WRAs’ eating habits related to their education status, with more than one-third of WRAs (38.5%) who had a primary education consuming food together with their family.

#### 3.2.3. Eating Practices Related to Religion and Culture

Specific foods were considered religious and/or cultural taboo foods. Pork meat is considered a taboo food in the broader society in the study areas, affecting the majority of WRA respondents (99.8%). In this study, (43.5%) of WRAs avoided goat milk while (10.2%) of WRAs avoided goat meat due to cultural reasons. Further, there are restrictions for women not to consume certain parts of chicken or lamb meat, due to culturally rooted gender inequality in the Amhara region [40]. Men are prioritized to consume these foods. In our study, more than one-third of WRAs (41.3%) accepted the norm that “women should not eat certain parts of chicken meat such as Feresegna/breasts, thighs and legs and lamb meat suck as kiltim (leg) (26.6%)”. Furthermore, (76.7%) of WRAs followed food restriction, which is religious fasting practice.

#### 3.2.4. Perception of Nutritious Diets

Less than half of WRAs (39.9%) were aware of the importance of consuming a variety of foods as part of a healthy diet, including ASF, vegetables, and fruit. More than one-third of WRAs (35.2%) were not aware of the components of a healthy diet. For example, (22.6%) of WRAs perceived a nutritious diet as eating ASF as the most important component, 3.3% (*n* = 14) as consisting of eating enough food regardless of its variety, and 9.3% to include the consumption of vegetables only, regardless of the consumption of other food groups. Beyond the above perceptions, about a quarter of WRAs (24.9%) stated that they have no information or knowledge about a nutritious diet.

### 3.3. Determinants of Dietary Diversity of WRA

The determinants of dietary diversity of WRAs are presented at the four levels of the socioecological framework: (i) intrapersonal/individual level; (ii) interpersonal/household level, (iii) community level, and (iv) institutional level. These are presented in the following sections and Table 3.

#### 3.3.1. Intrapersonal/Individual-Level Determinants

At the intrapersonal/individual level, our study identified six associated determinants: age, education, perception of a nutritious diet, frequency of consumption of ASF, and frequency of consumption of vegetables and fruit. A chi-square test showed that there was a statistically significant association between WRA and MDD-W (*p* = 0.036). Particularly, WRAs between 33–38 years were more likely than other WRAs to consume adequate MDD-W. The educational status of WRAs showed a statistically significant association with MDD-W (*p* < 0.001). WRAs having no education were more likely to have inadequate MDD-W, while WRAs who had attended primary and above education were more likely to have adequate MDD-W (*p* < 0.001). Moreover, WRAs who consumed ASF twice in a month achieved adequate MDD-W (*p* < 0.01) while WRAs who consumed ASF either only once a month or who never consumed ASF had inadequate MDD-W (*p* < 0.01). Furthermore, WRAs who consumed vegetables more frequently within a month were more likely to achieve adequate MDD-W (*p* < 0.001). Additionally, WRAs who consumed fruit at least once a week were more likely to achieve adequate MDD-W (*p* < 0.03) compared to WRAs who consumed fruit less frequently. WRAs who perceived a nutritious diet as consuming a variety of food were significantly associated with adequate MDD-W compared to their counterparts (*p* < 0.01), while WRAs who did not have knowledge of a nutritious diet were significantly associated with inadequate MDD-W (*p* < 0.04).

#### 3.3.2. Interpersonal/Household Level Determinants

At the interpersonal/household level, this study identified six determinants that showed statistically significant associations with MDD-W: the family’s actual eating occasion, husband’s education, women engaging in domestic tasks, women engaging in farming tasks, and decision-making power on food purchasing and consumption. With regard to the family’s actual eating occasions, specifically, WRAs who served food to their family before they consumed food had inadequate MDD-W, in comparison to WRAs who ate with their family (*p* < 0.03). WRAs whose husbands had attended primary and above education (*p* < 0.01) had adequate MDD-W. Moreover, WRAs who were responsible and engaged in domestic tasks without any support from their family had inadequate MDD-W (*p* < 0.046) compared to WAR who received support. Furthermore, WRAs who were involved in four or more farming tasks (i.e., soil preparation, weeding and harvesting, and animal rearing activities), indicating a high workload, had inadequate MDD-W (*p* < 0.03) compared to WRAs who were involved in less than three farming tasks, and who had adequate MDD-W (*p* < 0.01). The results of this study further revealed that WRAs who were involved in decision-making regarding food purchasing for the household had adequate MDD-W (*p* < 0.01), while WRAs had inadequate MDD-W if only their husbands were involved (*p* < 0.01). In households where both husband and wife decided on food consumption, WRAs had adequate MDD-W (*p* < 0.03).

#### 3.3.3. Community Level Determinants

At the community level, we identified six determinants showing statistically significant associations with MDD-W. One determinant related to the physical environment, specifically access to clean water. WRAs who had access to clean tap water were significantly associated with adequate MDD-W (*p* < 0.01), while WRAs who had access only to river and groundwater were more likely to have inadequate MDD-W (*p* < 0.01). Five determinants related to cultural beliefs on gender inequality and food consumption, expressed by Amharic proverbs and sayings, restricting and discouraging WRAs to consume adequate food and a variety of foods, and limiting their food preferences (i.e., what, how much and where to eat.) In the following, some examples are provided (translated by the first author from Amharic):

• ቅልጥም እና ፈረሰኛ ለአባወራ (k’lt’m ena feresegna leabawora): a girl/woman should not eat the main parts of the lamb or chicken, but these parts should rather be given to the husband/man;
• ሴት ልጅ መንገድ ላይ አትበላም (set liji menged lay atbelam): a woman should not eat on the street (in public);
• ከሴት ሆዳም የአንድ አመት በረዶ ይሻላል (keset hodam yand amet beredo yshalall): a seasonal disaster is better than a woman who is a voracious eater;
• የሴት ምራቋ ወፍራም ነው (yeset mɨrak’wa wefram new): a woman’s saliva is thick, so a woman cannot be hungry/she does not need to eat much, whereas a man is encouraged to eat a lot;
• እንኳን የሸመተ የአረሰም አይችልሽ (Enkuan yeshemete yearesem aychilish): no one can cover your food expenses as you are voracious eater (the woman). This proverb is mostly related to the manner of eating (i.e., WRAs should eat slowly, not eat in front of elders and guests, and should not eat much food)

Those WRAs who accepted and obeyed the above proverbs and sayings had inadequate MDD-W compared to WRA who did not accept the proverbs and sayings (*p* < 0.01).

#### 3.3.4. Institutions and Organisation Level

At the institutional level, religion was not significantly associated with MDD-W. However, fasting as a religious food restriction was significantly associated with MDD-W (*p* < 0.01).

## 4. Discussion

This study was conducted among WRAs in the Amhara region, West Gojjam and South Gonder zones in three rural kebeles to assess the trends of dietary behaviour and associated sociodemographic and sociocultural determinants of dietary diversity of WRAs. In this section, specific aspects of dietary behaviour, as well as key determinants of dietary diversity of WRAs, will be discussed.

### 4.1. Minimum Dietary Diversity Score (MDD-W)

More than a quarter of WRAs achieved the MDD-W score while the majority of WRAs had inadequate MDD-W. This means that the majority of WRAs consumed less than five food groups on the previous day, resulting in inadequate micronutrient intakes. Our findings are consistent with studies in Mali [41] and Burkina Faso [42] and specific regions in Ethiopia [43], where only 27%, 28%, and 27.7% of WRAs, respectively, achieved the MDD-W. Our findings are lower than those reported in Nigeria [44], Uganda [45], and selected regions of Ethiopia [20], with adequate MDD-W ranging from 44–88.3%. Possible explanations for these discrepancies may include differences in rural/urban influences [45], socioeconomic status [45], differences in agroecological conditions [27], seasonal variations [27], as well as cultural and religious differences [27].

Assessment of dietary diversity showed that WRAs consume a diet consisting mainly of starchy staples such as grains (e.g., teff, dagusa/finger millet, maize, and wheat) and roots and tubers (e.g., potato). Few WRAs included ASF, dark green leafy vegetables, other vitamin A-rich fruit and vegetables, and other fruit (e.g., banana and avocado) in their diet. The predominant consumption of starchy staples in our study is consistent with other studies from Ethiopia [20], Mali [41], Nigeria [44], and Kenya [19] where the vast majority of women consumed starchy staples. Dark green leafy vegetables and other fruit were also less consumed food groups by WRAs in Mali [41]. The higher consumption of starchy staples, roots, tubers, and pulses in our study can partly be explained by food habits/culture, which will be discussed in detail in the next paragraph. Another explanation, closely linked with food habits, is the fact that these staple crops dominate the agricultural production in the Amhara region, while vegetables, except for onion and tomato, are less cultivated [20,46]. Similar to our findings, a study in Kenya showed that meat/poultry/fish and eggs were the least consumed food groups by WRAs [19], while in Nigeria a higher proportion (77%) of WRAs consumed meat/fish/poultry [44]. The variation in the results could be due to sociocultural differences and also due to differences in urban and rural settings. In Ethiopia, unlike Nigeria, the majority of the population is Orthodox Christian, resulting in different religious dietary practices. Evidence from other studies in Ethiopia shows that meat is mostly consumed during holidays and special festive occasions [27,47,48]. In our study area, ASFs are expensive, as is the case in many other parts of Ethiopia, and, mostly, there is no butcher in rural areas [47]. In the Nigerian study [44], sampling included both urban and rural settings, which can result in variance regarding dietary diversity. Additionally, in Nigeria, the majority of women were educated, different from our study. Thus, educated women could be economically and socially more empowered and might have greater access to and control over resources, enhancing more diversified diets [19].

### 4.2. Food Habits

With regard to eating habits as another aspect of dietary behaviour, the findings of this study show that more than half of WRAs gave priority to their husbands or other family members in terms of food consumption. Few women ate before feeding their families. The eating habits within the family reveal that this is largely determined by sociocultural practices where other family members, particularly men, receive priority over women regarding food allocation and consumption, resulting in gender disparity. This has been confirmed by other studies in Ethiopia [40,49] but has also been observed in studies in India [50], Nepal [51,52], and Burkina Faso [53], where women usually ate last due to cultural norms and even ate the leftovers or did not eat at all if the food was limited.

### 4.3. Food Restrictions

In most parts of Ethiopia, the consumption of meat and dairy products is limited to some domestic animals such as cow, ox, sheep, goat, and chicken [47,48], while pork and also donkey and horse meat [48] are not allowed for human consumption. Our study confirmed that pork meat is generally restricted for consumption due to religious and cultural beliefs. Moreover, our study observed a trend of restricting WRAs from eating certain parts of chicken, as has been observed in other parts of Ethiopia [40], or lamb meat. Our results further show that a substantial number of WRAs (*n* = 183, 43.5%) avoided goat milk and a smaller number avoided goat meat. A study conducted in the northern part of Ethiopia revealed that goat meat is associated with bad spirits, which can cause sickness and is, therefore, considered a taboo food [48]. Whether food is regarded as taboo in Ethiopia depends on geographical location related to availability and cultural and religious practices. For instance, the consumption of camel milk and meat is common among the pastoralist communities in the eastern parts of Ethiopia, while these foods are taboo in other parts of Ethiopia, including the Amhara region [48]. Even though the majority of WRAs in our study did not practice food taboos except for pork meat, our findings show that food taboos still exist, posing challenges for the diet quality and the health of WRAs, as has been confirmed by other authors [48]. These kinds of food restrictions and dietary behaviours might hinder the dietary diversity of women.

### 4.4. Determinants of Dietary Diversity at Intrapersonal/Interpersonal/Community/Institutional Level

The dietary diversity of WRAs is one of the most important elements for positive lifelong and intergenerational nutritional status [2,54]. This section discusses selected determinants affecting WRAs’ dietary diversity in rural Ethiopia, at the different levels of the socioecological framework.

Determinants at the intrapersonal/individual level that show significant associations with women’s MDD-W include age, education, perception of nutritious diet, frequency of consumption of ASF, vegetables, fruit, and residence. Evidence shows that education is key to women’s wellbeing and to reducing infant mortality [55]. In this study, WRAs who had attended school were more likely to achieve MDD-W than WRAs who were not able to read and write. This is consistent with studies in Kenya [19], Uganda [45], and different parts of Ethiopia [20,55]. Women’s education is key to bringing changes not only for women’s lives but also for their families and the communities as a whole [56].

At the interpersonal/household level, the husband’s education, the family’s actual eating occasion/habit, women engaged in domestic and farming tasks, and decision-making power on food purchasing and food consumption were significantly associated determinants of MDD-W. The husband’s education enhances adequate MDD-W. Similar to our findings, studies in different parts of Ethiopia found that a husband with a diploma and higher educational status is positively associated with pregnant women’s dietary practices [57], whereas an uneducated husband is associated with pregnant women’s food aversion that could affect women’s dietary diversity negatively [58,59].

Ethiopian society is still structured according to patriarchal norms. Women’s activities are mostly limited to the home and taking care of cooking, bearing and rearing children, as well as being involved in farming tasks [43,60,61]. Our study shows that high workloads on women, such as performing domestic tasks without support from their family and, additionally, engaging in four or more farming tasks resulted in inadequate MDD-W. A review of the literature in developing countries reported that women contribute to more than 40% of agricultural labour in 52 African countries [62]. A qualitative study in India revealed that workload and social and cultural norms were among the factors that contributed to inadequate intake of vegetables and fruit of WRA [63]. This is also supported by a study in other parts of Ethiopia [29]. This implies that the workload has a negative effect on the dietary diversity of WRAs. It is obvious that women carry the burden of almost all household responsibilities as well as multiple tasks in farming. Thus, the sharing of domestic and farm labour (i.e., culturally defined as female and male roles, respectively) could be one way to reduce the workload of WRAs that affects the dietary behaviour and diet quality of WRAs. The socioecological framework illustrates the close interlinkage of all levels. Patriarchal structures and the resulting sociocultural norms influence individual behaviour, household dynamics, and interactions at the community level. This has an impact on women’s workloads and access to various resources. Therefore, the different levels in the socioecological framework are interlinked and cannot be separated.

As presented in the result section, the decision-making power of women is another important determinant at the interpersonal/household level that affects the dietary diversity of WRA. Our findings show that women who were not involved in decision-making regarding food purchasing and consumption had inadequate MDD-W compared to those women who were involved in decision-making. This is in line with studies from Ghana [64], India [63], and different parts of Ethiopia [20,43]. Thus, women’s decision-making power increases their access to a greater variety of foods, improving their and their children’s dietary diversity. A qualitative study in India shows that women’s lack of participation in food purchasing and consumption decisions is due to the cultural norms and beliefs of communities that portray women as dependent and of low social status, while men/husbands are accorded higher social status and privileges [63]. Several studies provide evidence that empowering women is key for improving access to household resources and decision-making power in general and for improved dietary diversity of women and their children in particular [63,64,65].

At the community level, access to clean water and cultural beliefs are determinants associated with MDD-W in our study. Access to basic infrastructure such as clean water, sanitation, and hygiene is key to good health outcomes [66]. In Ethiopia, just under half (47.3%) of rural households have access to a basic drinking water supply, i.e., access to drinking water from an improved source and 30 min or less to collect it [35]. Women and girls are responsible for household water collection, storage, and treatment of water in the household but are disproportionally affected by poor access to water, sanitation, and hygiene services, and are often not involved in community water point planning and construction [66]. Moreover, according to UNICEF, 60% to 80% of communicable diseases and 50% of undernutrition among children in Ethiopia are due to poor hygiene and lack of access to a safe water supply [67]. In Ethiopia, access to improved drinking services, i.e., tap water, is largely linked to socioeconomic status [35]. Our study shows that WRAs who use tap water are more likely to have adequate MDD-W. Having access to tap water means that clean water is available for cooking and drinking. It also means less time burden for women to fetch water and being less exposed to health risks. Similarly, a study in the Tigray region showed that lactating mothers using protected well water were four times more likely to have inadequate dietary diversity and also had lower socioeconomic status than women using tap water [68]. Social inclusion in the design and construction of water, sanitation and hygiene services, as well as promoting dialogues at the household and community level with men and boys about household chores and decision-making power are key to improving women’s and girls’ access to water, sanitation, and hygiene, and the associated health outcomes for women and children [67].

Cultural practices related to food play an important role in shaping communities’ diets, food preferences, and intrahousehold food allocation and distribution [34,43,63]. There are various commonly used Amharic proverbs and sayings reflecting gender inequality with regard to food consumption. In this study, we focus on the common use of Amharic proverbs and sayings that are significantly associated with MDD-W. As presented in the results section, these proverbs and sayings give priority to men with regard to food allocation and pose several restrictions on women; for example, not to eat on the street/in public, not to eat adequate amounts of food, and not to eat excessively/being voracious eaters. This might be a result of wrong perceptions about women. A study in Ethiopia argues that, as women are engaged in domestic chores for a large part of the day, it is wrongly assumed that they are able to eat whatever food is available and, therefore, they are considered voracious eaters [43,69,70]. These misperceptions and cultural norms lead to gender-based discrimination with regard to food consumption, hindering women from consuming adequate nutritious diets, also described as ‘food violence’ [71]. Unless the larger society abolishes such kinds of proverbs and sayings, efforts to support healthy dietary behaviour and women’s empowerment will have only a limited impact. Ethiopia signed the Convention on the Elimination of all Forms of Discrimination against Women (CEDAW) in 1980 [72]. Therefore, the government and concerned institutions in Ethiopia are obliged to prevent gender-based discrimination in all its forms and to adopt more culturally sensitive approaches to support the healthy dietary behaviour of WRAs.

At the broader institutions/organisation level, even though there are different determinants of MDD-W we emphasise here to understand the relationship between religion as one institution with MDD-W in the Amhara region. Our study shows that religion had no significant association with MDD-W. However, related to religious dietary practices, the majority (76.7%) of WRAs in our study practised religious fasting. Similar to our findings, a qualitative analysis in different parts of Ethiopia identified that fasting was one of the barriers that affects the dietary practice of pregnant women [29] and children [24]. In contrast, a study in Bangladesh reported that during the Ramadan fasting period, women, all of whom were Muslim, had higher dietary diversity than during nonfasting periods [73]. These different outcomes might be due to the fact that, in our study, nearly all participants (91.4%) were Orthodox Christians. During fasting, there are severe restrictions regarding the types of food that can be consumed. For example, no ASFs are allowed. There are also restrictions regarding the frequency of meals, usually having one meal per day in the afternoon or early evening. Unlike the Orthodox Christian religion, fasting in Islam is not posing restrictions regarding the types of foods consumed, but no food or liquids are to be consumed from sunrise to sunset. After sunset and before sunrise food intake is allowed, with no restrictions [48].

Religious fasting practice is one of the eating behaviours shaped by the society’s religious doctrine who follow a particular religion affecting the diet quality and MDD-W [24,47,48]. Therefore, attention should be given to educating women about finding alternative options, such as replacing ASFs during the fasting period with equivalent non-animal-sourced food groups that contain similar nutrients. Further, broad awareness should be created about the significance of a nutritious diet for the health of WRAs, as well as for their children, during and outside of the fasting periods. Furthermore, family is fundamental to bringing structural or ideological change within the community, as well as the whole society. Thus, the government and other public sectors should design a strategy to improve diet quality, especially during the fasting season by encouraging women, family members, neighbours, especially highly respected elders, and religious leaders. Family and close friends, neighbors and respected people have a great influence on individual food preference and adequacy [48]. Our results could be a starting point for more detailed studies on Amharic proverbs and sayings related to the food intake of women and their negative effects not only on dietary diversity but also on other aspects of women’s lives.

*Strengths and Limitations*

A strength of this study is that it pays attention to the social and cultural determinants affecting MDD-W, in addition to individual and sociodemographic characteristics. Even though there are studies that identified similar cultural practices such as taboo foods [26,27,28,29], religious fasting [24,25,47], and decision-making power of women [20,21,43] as determinants of dietary diversity among pregnant women and WRAs, this study is the first to assess dietary behaviours and their association with cultural beliefs, specifically the impact of the use of Amharic proverbs and sayings on dietary diversity of WRAs.

This study also has some limitations. It draws on a single qualitative 24 h recall of dietary diversity but did not assess the routine dietary practice of WRAs over a longer period. Dietary data are based on self-reporting and might be subject to sociocultural desirability bias and recall bias. Further, our data do not enable us to determine causal relationships. Moreover, our findings cannot be generalized for the dietary practices of WRAs in Ethiopia due to the sampling strategy. Data on dietary practices were collected after the harvesting season and therefore do not represent different seasons throughout the year. Moreover, it would have been beneficial if the study was supported by a qualitative study to address different sociocultural food practices, especially to gain detailed information about the magnitude of the impact of proverbs and sayings on the dietary behaviour of WRAs.

## 5. Conclusions

This study assessed different dietary behaviours and associated determinants of dietary diversity of WRA. The majority of WRAs participating in this study had inadequate MDD-W, which refers to inadequate micronutrient intake, affecting the health conditions of both WRAs and their children. Our study reveals that the dietary behaviour of WRAs was mostly linked with their cultural and religious practices, associated with fasting, lack of decision-making power, and high workload of women. Moreover, our study found different associated factors based on the socioecological framework, such as the use of proverbs and sayings, resulting in gender-based discrimination and inequality with regard to food consumption, negatively affecting the dietary diversity of WRAs and their children. To overcome the inadequate dietary diversity of WRAs, in addition to individual-level determinants, attention and priority should be given to sociocultural determinants at the interpersonal/household level, which are strongly linked to gender inequality regarding food consumption and decision-making power. At the community level, determinants such as cultural beliefs related to taboo foods and food restrictions using Amharic proverbs and sayings that are still widely being practiced, negatively affect the dietary diversity of WRAs. Our study added Amharic proverbs and sayings as a novel determinant to the socioecological framework. This determinant was not studied before as a means of addressing gender inequality related to food consumption. This model corroborates the need for more culturally sensitive and adapted approaches when investigating the dietary behaviour of WRAs. Empowering women in all economic, social, and cultural aspects should be a priority to improve not only the nutritional status of women and their children but also the overall family’s dietary behaviour and health conditions.

## Figures and Tables

**Table 1:** Sociodemographic characteristics of WRA (*n* = 421).

Variables	Frequency (n)	Percent (%)
Age (in years)		
<=21	58	13.5
22–25	68	15.8
26–28	65	15.1
29–32	133	30.9
33–38	60	13.9
39+	46	10.7
Religion		
Orthodox	385	91.4
Muslim	36	8.6
No religion	-	-
Residence		
Korata rural kebele (South Gonder zone)	239	55.5
Kolela rural kebele (West Gojjam zone)	107	24.8
Meshenti rural kebele (West Gojjam zone)	75	17.4
Education: Women		
Not able to read and write	171	40.6
Able to read and write	128	30.4
Primary education (G1-8)	122	29.0
Secondary education (G9-12)	-	-
Education: Husbands		
Not able to read and write	134	31.8
Able to read and write	135	32.1
Primary education (G1-8)	106	25.2
Secondary education (G9-12)	39	9.3
Higher education	7	1.7
Source of HH income		
Agriculture	250	59.4
Nonagriculture	150	35.6
Agriculture and others	21	5.0
Household size (number of children)		
1–3	232	55.1
4–6	172	40.9
>=7	84	4.0

Source: Original data collected in our survey on sociodemographic characteristics of WRA in Amhara region, 2019.

**Table 2:** Dietary behaviour of WRAs in Amhara region, Ethiopia (*n* = 421).

Dietary Behaviours	Total *n* = 421	Percent (%)
Eating habits		
Family’s actual eating occasion		
Food intake together with the family	157	37.3
Food intake after serving the family	252	59.9
Food intake before serving the family	12	2.9
Religious eating practice (fasting)		
All fasting days	323	76.7
Major fasting days, Wednesday and Friday	71	16.9
I do not fast	27	6.4
Cultural eating practices (taboo foods and food restrictions)		
Taboo foods		
Pork meat	420	99.8
Goat meat	43	10.2
Goat milk	183	43.5
Food restrictions		
Lamb meat (some parts like ‘kiltim’ ‘firimba’)	112	26.6
Chicken meat such as Feresegna, breasts, thighs and legs	174	41.3
Prioritize husbands for food intake	175	41.6
Perception of a nutritious diet		
Consumption of a variety of foods	168	39.9
Consumption of animal-source food	95	22.6
Consumption of vegetables	39	9.3
Consumption of food until full	14	3.3
I do not know	105	24.9

Source: Original data collected in our survey on characteristics of dietary behaviours of WRA in Amhara region, 2019.

**Table 3:** Minimum dietary diversity and determinants among WRA based on the socioecological conceptual framework.

Variables	MDD-W Inadequate N (%)	MDD-W Adequate N (%)	Chi-Square	*p*-Value
Intrapersonal level determinants				
Sociodemographic determinants				
Age of the respondents (in years)				
<=21	41 (71.9)	16 (28.1)		
22–25	48 (71.6)	19 (28.4)		
26–28	49 (76.6)	15 (23.4)		
29–32	101 (78.3)	28 (21.7)		
33–38	32 (56.1)	25 (43.9)	9.99	* 0.01
39+	9 (80.4)	37 (19.6)		
Education of the women			34.579	* 0.001
Not able to read and write	148 (86.5)	23 (13.5)	26.32	
Able to read and write	92 (71.9)	36 (28.1)		
Primary (G1-8) and higher	68 (55.7)	54 (44.3)	26.52	* 0.01
Household source of income			1.859	0.395
Agriculture	180 (72.0)	70 (28.0)		
Nonagriculture	110 (73.3)	40 (26.7)		
Agriculture and others	23 (74.2)	8 (25.8)		
Residence				
Kolela rural kebele	87 (81.3)	20 (18.7)	5.38	* 0.02
Korata rural kebele	168 (70.3)	71 (29.7)		
Meshenti rural kebele	53 (70.7)	22 (29.3)		
Food and nutrition knowledge				
Perception of nutritious diet				
Eat until full	10 (71.4)	4 (28.6)		
Eat animal source foods	76 (80.0)	19 (20.0)		
Eat variety of foods	104 (61.9)	64 (38.1)	18.06	* 0.01
Eat vegetables	33 (84.6)	6 (15.4)		
I don’t know	85 (81.0)	20 (19.0)	4.33	* 0.04
Food habit				
Frequency of consumption of ASF				
Once a week	19 (61.3)	12 (38.7)		
Twice a week	7 (38.9)	11 (61.1)		
Twice a month	35 (62.5)	21 (37.5)		
Once a month	87 (69.6)	38 (30.4)	13.76	* 0.01
During holidays	147 (82.6)	31 (17.4)	13.76	* 0.01
Never eat	13 (100)	0 (00)		
Frequency of consumption of vegetables				
Every Day	9 (39.1)	14 (60.9)	14.36	* 0.001
Once a week	108 (69.2)	48 (30.8)		
Twice a week	39 (72.2)	15 (27.8)		
Once a month	137 (79.7)	35 (20.3)	6.25	* 0.01
Never eat	15 (93.8)	1 (6.3)		
Frequency of consumption of fruit				
Once a week	56 (58.3)	40 (41.7)	13.91	* 0.001
Twice a week	20 (74.1)	7 (25.9)		
Once in a month	178 (78.4)	49 (21.6)	6.92	* 0.01
Never eat	54 (76.1)	17 (23.9)		
Interpersonal/Household-level determinants				
Household environment				
Household size			0.079	0.779
1–3 children	171 (73.7)	61 (26.3)		
4–8 children	137 (72.5)	52 (27.5)		
Household composition				
Husband education				
Not able to read and write	103 (76.9)	31 (23.1)		
Able to read and write	105 (77.8)	30 (22.2)		
Primary (G1-8) and higher	100 (65.8)	52 (34.2)	6.60	* 0.01
WRA engaging in domestic tasks				
Domestic tasks without family support	134 (78.4)	37 (21.6)	3.971	* 0.046
Domestic tasks with family support	174 (69.6)	76 (30.4)		
WRAs engaging in farming tasks				
1–3 tasks	3 (33.3)	6 (66.7)	7.45	* 0.01
4–5 tasks	58 (84.1)	11 (15.9)	4.97	* 0.03
6 or more tasks	247 (72.0)	96 (28.0)		
WRAs decision to purchase food				
Wife	69 (63.3)	40 (36.7)	7.29	* 0.01
Husband	86 (83.5)	17 (16.5)	7.40	* 0.01
Both	153 (73.2)	56 (26.8)		
WRAs decision to consume food				
Wife	209 (75.2)	69 (24.8)		
Husband	16 (94.1)	1 (5.9)	3.96	0.05
Both	83 (65.9)	43 (34.1)	4.84	* 0.03
Social environment				
Family actual eating occasion				
Food intake together with the family	170 (68.2)	50 (31.8)		
Food intake after serving the family	194 (77.0)	58 (23.0)	4.67	* 0.03
Food intake before serving the family	7 (58.3)	5 (41.7)		
Community-level determinants				
Physical environment				
Access to clean water				
River, tap water, and groundwater	32 (74.4)	11 (25.6)		
River and tap water	6 (85.7)	1 (14.3)		
River and groundwater	42 (89.4)	5 (10.6)	7.08	* 0.01
Tap water and groundwater	106 (70.7)	44 (29.3)		
Tap water	37 (58.7)	26 (41.3)	7.84	* 0.01
Groundwater	85 (76.6)	26 (23.4)		
Energy for cooking			1.856	0.395
Firewood	165 (72.2)	65 (29.8)		
Firewood and manure	111 (76.6%)	34 (23.4)		
Manure	28 (66.7)	14 (33.3)		
Cultural belief				
Socially constructed Amharic proverbs				
Prioritize husbands for food consumption ቅልጥም እና ፈረሰኛ ለአባወራ WRAs who accept WRAs who do not accept	248 (82.9) 60 (49.2)	51 (17.1) 62 (50.8)	50.27	* 0.01
A woman should not eat on the street (in public), ሴት ልጅ መንገድ ላይ አትበላም WRAs who accept WRAs who do not accept	210 (79.2) 98 (62.8)	55 (20.8) 58 (37.2)	13.47	* 0.01
Women should not eat much food (voracious eater) ከሴት ሆዳም የአንድ አመት በረዶ ይሻላል WRAs who accept WRAs who do not accept	173 (82.0) 135 (64.3)	38 (18.0) 65 (35.7)	16.81	* 0.01
Manner of eating (eating slowly, not eating in front of elders and guests) እንኳን የሸመተ የአረሰም አይችልሽ WRAs who accept WRAs who do not accept	150 (80.6) 158 (67.2)	36 (19.4) 77 (32.8)	9.49	* 0.01
Culturally rooted food restriction Women restricted to certain parts of chicken and lamb meat Yes No	248 (82.9) 60 (49.2)	51 (17.1) 62 (50.8)	50.29	* 0.001
Institutions and organisation-level determinants				
Religion			0.277	0.599
Orthodox Christian	283 (73.5)	102 (26.5)		
Muslim	25 (69.4)	11 (30.6)		
Religious fasting practice				
All fasting days	247 (76.5)	76 (23.5)	7.73	* 0.01
Major fasting days and Wednesday and Friday	54 (76.1)	17 (23.9)		
Do not fast	7 (25.9)	20 (74.1)	32.83	* <0.01

Chi-square test, * statistically significant association at *p* < 0.05, determinants of dietary diversity of WRA.

## Data Availability

The data presented in this study are available on request to the corresponding author.
